# New Records of* Hyphoderma* (Meruliaceae, Polyporales) for India

**DOI:** 10.1155/2017/3437916

**Published:** 2017-02-06

**Authors:** Sanjeev Kumar Sanyal, Ritu Devi, Gurpaul Singh Dhingra

**Affiliations:** ^1^Department of Botany, Punjabi University, Patiala 147002, India; ^2^ICAR, Directorate of Mushroom Research, Chambaghat, Solan 173213, India

## Abstract

An account of eight species of genus* Hyphoderma* (*H*.* clavatum*,* H*.* definitum*,* H*.* echinocystis*,* H*.* litschaueri*,* H*.* nemorale*,* H*.* subpraetermissum*,* H*.* tibia,* and* H*.* transiens*) is presented, which is based on collections made from Uttarakhand state during 2009–2014. All these species are cited and fully described for the first time from India.

## 1. Introduction

The genus* Hyphoderma* Wallr. is a large and heterogeneous assemblage of species in Agaricomycetes, kept together on the basis of generally large sized, smooth basidiospores and constricted basidia, rich in both oil drops and hyphae with conspicuous clamps. It was proposed by Wallroth [1] with* H*.* spiculosum* Wallr. as the type species. Later, it was emended by Donk [2], who described the genus with* Thelephora setigera* Fr. as the type species. Both Eriksson [3] and Parmasto [4] followed the emended version given by Donk [2] in general, except division of the genus into sections and subsections by Parmasto. Eriksson and Ryvarden [5] made some changes to the system of Parmasto [4] and made twelve groups to describe twenty seven species from North Europe. In 1976, they added two more species from North Europe to the earlier list. Maekawa [6], Larsson [7], and Bernicchia and Gorjón [8], on the basis of molecular studies, made delimitations within the genus. But keeping in view the incomplete molecular work, in the present study, the traditional concept of Donk [2] with emendations made by Parmasto [4] and Eriksson and Ryvarden [5] has been followed. The genus is distributed worldwide with 130 published species (http://www.mycobank.org). Earlier, from India, 36 taxa have been reported/listed by the different workers [9–23] from different localities. This paper provides information about eight species of genus* Hyphoderma*, all of which constitute new records for India. A key to all the taxa reported from Uttarakhand has been given.

## 2. Materials and Methods

Specimens have been collected from the various localities of the Uttarakhand during the various fungal forays conducted from 2009 to 2014. Microscopic details related to various structures, that is, hyphae, cystidia, basidia, and basidiospores of the specimens, were studied by making crush mounts and hand cut sections in water, 3–5% KOH solutions and staining in various reagents like Congo red, Phloxine, Cotton Blue, Melzer's Reagent, and Sulphovanillin. Line diagrams were made by using camera lucida attached to the compound microscope at various magnifications and lense combinations. Color standards were used as per Mathuen's Handbook of Color by Kornerup and Wanscher [24]. Scale has been provided on the macro images (10 bars are equivalent to 1 cm). Specimens have been deposited in the Herbarium of Department of Botany, Punjabi University, Patiala, India (PUN). Nomenclature follows Blackwell et al. [25], James et al. [26], Hibbett et al. [27], Kirk et al. [28], Bernicchia and Gorjón [8], and http://www.mycobank.org as far as possible.

## 3. Study Area

Uttarakhand is the 27th state of the Republic of India, situated in the Northern part of India between 28°43′N to 31°28′N latitude and 77°34′E to 81°03′E longitude. It became the 27th state of Republic of India on 9th November 2000 and has a total geographical area of 53,483 km^2^. The recorded forest area of the state is 34,651 km^2^ which constitutes 64.79% of its geographical area, (reserved forests 71.11%, protected forests 28.52%, and unclassed forests 0.35%). It has Tibet on its North, Nepal on its East, Uttar Pradesh to its South, Haryana to its West, and Himachal Pradesh to its North-West (http://www.fsi.org.in). The state is divided into 2 divisions, that is, Kumaon division and Garhwal division. Kumaon division has 6 districts, Almora, Bageshwar, Champawat, Nainital, Pithoragarh, and Udham Singh Nagar, whereas Garhwal division has 7 districts, Chamoli, Dehradun, Haridwar, Pauri Garhwal, Rudraprayag, Tehri Garhwal, and Uttarkashi. In hilly region, the climate in general is cold and humid and of temperate type but varies with altitude. The different zones are identified as warm temperate (900 m–1800 m), cool temperate (1800 m–2400 m), cold zone (2400 m–3000 m), alpine zone (3000 m–4000 m), glacier zone (4000 m–4800 m), and perpetually frozen zone (above 4800 m). The valleys are hot in summer and much colder in winter (http://www.fsi.org.in).

## 4. Taxonomy

**Figure 1:**
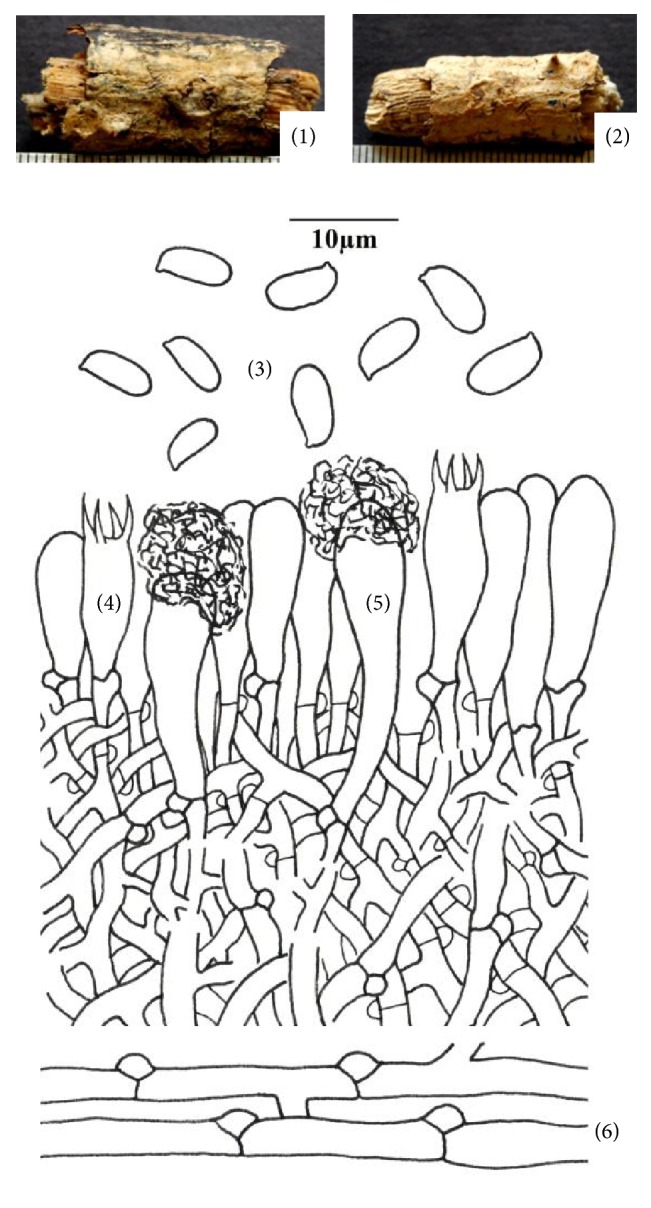
*Hyphoderma clavatum* (1)–(6). (1) Fresh basidiocarp, (2) dried basidiocarp, (3) basidiospores, (4) basidium, (5) cystidium, and (6) generative hyphae.

*(1) Hyphoderma clavatum Sheng H. Wu, Botanical Bulletin of the Academia Sinica (Taipei) 38: 64, 1997 (Figure 1(1)–(6)).* Basidiocarp resupinate, adnate, effused, up to 250 *μ*m thick in section; hymenial surface smooth, yellowish white to pale yellow when fresh, pale orange to orange white on drying, margins thinning, paler concolorous, to indeterminate. Hyphal system monomitic. Generative hyphae branched, septate, thin-walled, clamped; basal hyphae up to 3.8 *μ*m wide, parallel to substrate, loosely interwoven; subhymenial hyphae up to 3.0 *μ*m wide, vertical, compact. Cystidia 11.8–28.0 × 3.8–5.6 *μ*m, narrowly clavate to subcylindrical, capitate, apically encrusted, with basal clamp; enclosed in the hymenium. Basidia 12.4–18.8 × 4.4–5.6 *μ*m, clavate, 4-sterigmate, with clamp; sterigmata up to 3.8 *μ*m long. Basidiospores 6.2–8.0 × 2.4–3.8 *μ*m, ellipsoid, smooth, thin-walled, acyanophilous, inamyloid.

*Specimen Examined.* India, Uttarakhand: Dehradun, Jabarkhet, on stick of* Quercus leucotrichophora* A. Camus, Sanyal 6672 (PUN), August 20, 2010.

*Remarks. Hyphoderma clavatum* is characterized by being resupinate, adnate, effused, smooth yellowish white to pale yellow basidiocarp, narrowly clavate to subcylindrical, capitate, and apically encrusted cystidia with ellipsoid basidiospores. Wu [29] reported it from Taiwan. However, it is being reported for the first time from India.

**Figure 2:**
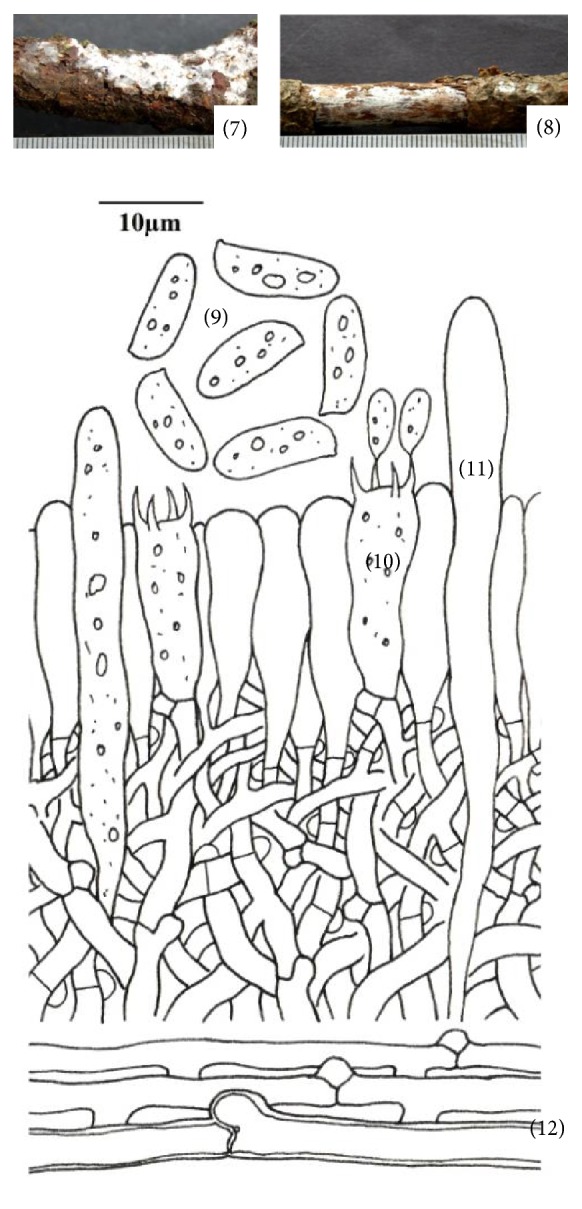
*Hyphoderma definitum* (7)–(12). (7) Fresh basidiocarp, (8) dried basidiocarp, (9) basidiospores, (10) basidium, (11) cystidium, and (12) generative hyphae.

*(2) Hyphoderma definitum (H.S. Jacks.) Donk, Fungus 27: 15, 1957. – Corticium definitum H.S. Jacks., Canadian Journal of Research 26 (2): 149, 1948 (Figure 2(7)–(12)).* Basidiocarps resupinate, adnate, effused, up to 100 *μ*m thick in section; hymenial surface smooth, pruinose, grayish white when fresh, not changing much on drying; margins thinning, paler concolorous, to indeterminate. Hyphal system monomitic. Generative hyphae up to 4.2 *μ*m wide, branched, septate, clamped; basal hyphae thin- to somewhat thick-walled, forming a loose texture; subhymenial hyphae thin-walled, compact. Cystidia 51.0–70.0 × 4.4–5.8 *μ*m, subcylindrical to subfusiform, thin-walled, with basal clamp, with or without oily contents. Basidia 18.0–20.2 × 5.8–6.8 *μ*m, clavate, 4-sterigmate, with basal clamp and oily contents; sterigmata up to 4.4 *μ*m long. Basidiospores 10.6–12.2 × 3.8–4.2 *μ*m, cylindrical, smooth, thin-walled, acyanophilous, inamyloid, with oily contents.

*Specimens Examined.* India, Uttarakhand: Tehri Garhwal, Jaunpur, on stick of* Quercus leucotrichophora* A. Camus, Sanyal 6674 (PUN), August 20, 2010; Tehri Garhwal, Jaunpur, on stick of* Quercus leucotrichophora* A. Camus, Sanyal 6675 (PUN), August 20, 2010.

*Remarks.* This species is characterized by being grayish white basidiocarps, subcylindrical to subfusiform cystidia and cylindrical basidiospores. The species was first described by Jackson in 1948 [30] as* Corticium definitum* H. S. Jacks. Donk [2] shifted it to genus* Hyphoderma*. It has earlier been reported from Finland, Norway, Scandinavia and Sweden (http://www.mycobank.org). Here, it is being described for the first time from India.

**Figure 3:**
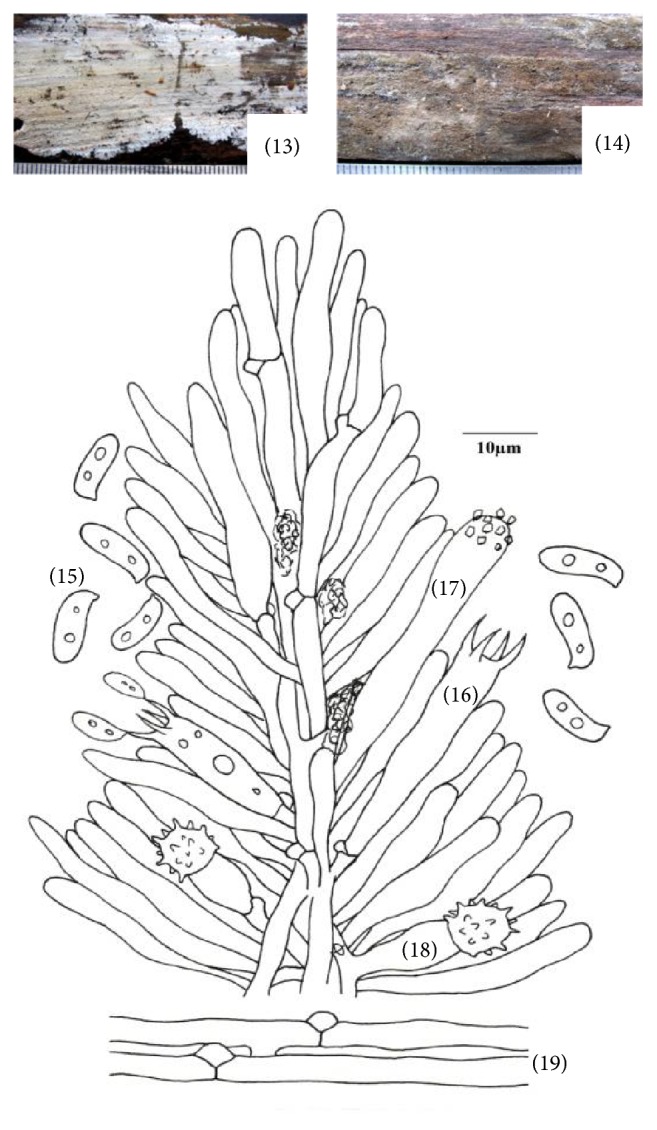
*Hyphoderma echinocystis* (13)–(19). (13) Fresh basidiocarp, (14) dried basidiocarp, (15) basidiospores, (16) basidium, (17) cystidium, (18) echinocyst, and (19) generative hyphae.

*(3) Hyphoderma echinocystis J. Erikss. & Å. Strid, The Corticiaceae of North Europe 3: 471, 1975 (Figure 3(13)–(19)).* Basidiocarp resupinate, effused, adnate, up to 230 *μ*m thick in section; hymenial surface finely odontoid, pale yellow to grayish yellow when fresh, not changing much on drying; margins thinning, paler concolorous, to indeterminate. Hyphal system monomitic. Generative hyphae up to 4.0 *μ*m wide, branched, septate, clamped, thin-walled; basal hyphae parallel to the substrate, loosely arranged, with brownish excreted matter; subhymenial hyphae vertical, denser. Numerous thin-walled, echinulate cells (echinocysts) up to 7.4 *μ*m wide present in the subhymenium. Excretory brownish amorphous matter present in the context. Cystidia 34.0–42.0 × 5.6–6.2 *μ*m, hyphoid, subclavate to subcylindrical, thin-walled, with brown excrete at the apex. Basidia 16.0–28.0 × 5.6–6.8 *μ*m, clavate, 4-sterigmate, with basal clamp, with or without oily contents; sterigmata up to 4.8 *μ*m long. Basidiospores 8.6–10.6 × 3.0–3.8 *μ*m, suballantoid to allantoid, thin-walled, smooth, acyanophilous, inamyloid, with oily contents.

*Specimen Examined.* India, Uttarakhand: Pauri Garhwal, Adwani, on log of* Pinus roxburghii* Sarg., Sanyal 6677 (PUN), July 23, 2011.

*Remarks.* This species is characterized by being finely odontoid, pale yellow to grayish yellow basidiocarp, and suballantoid to allantoid basidiospores and by the presence of echinocysts in subhymenium. It was first described by Eriksson and Strid [31] from Sweden. It has earlier been reported from America, Europe, Iran, Japan, and Taiwan (http://www.mycobank.org). Here, it is being reported as new record for India.

**Figure 4:**
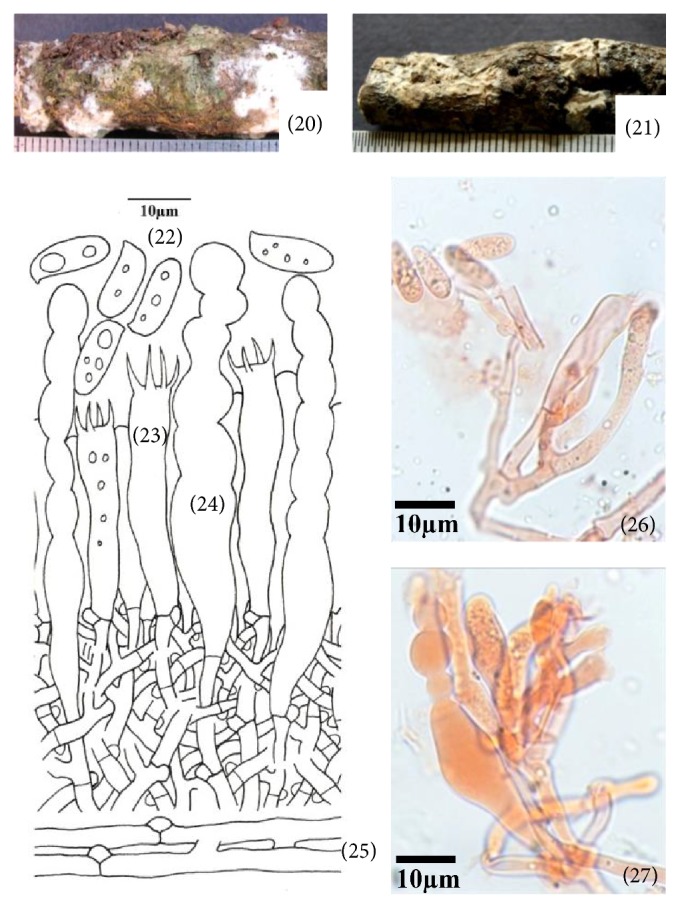
*Hyphoderma litschaueri* (20)–(27). (20) Fresh basidiocarp, (21) dried basidiocarp, (22) basidiospores, (23) basidium, (24) cystidium, (25) generative hyphae, (26) photomicrographs showing basidiospores and generative hyphae, and (27) photomicrographs showing cystidium and generative hyphae.

*(4) Hyphoderma litschaueri (Burt) J. Erikss. & Å. Strid, The Corticiaceae of North Europe 3: 481, 1975. – Corticium litschaueri Burt, Annals of the Missouri Botanical Garden 13 (3): 259, 1926 (Figure 4(20)–(27)).* Basidiocarp resupinate, adnate, effused, up to 170 *μ*m thick in section; hymenial surface smooth to tuberculate, grayish white when fresh, orange white to pale orange on drying; margins thinning, byssoid, paler concolorous, to indeterminate. Hyphal system monomitic. Generative hyphae up to 3.8 *μ*m wide, thin-walled, branched, septate, clamped; basal hyphae loose, parallel to the substrate; subhymenial hyphae compact, vertical. Cystidia 65.0–79.0 × 6.2–10.0 *μ*m, clavate, constricted to moniliform towards the apex, thin- to somewhat thick-walled, with basal clamp. Basidia 30.0–40.0 × 6.2–7.2 *μ*m, clavate to subclavate, 4-sterigmate, with basal clamp; sterigmata up to 6.8 *μ*m long. Basidiospores 12.4–13.8 × 4.8–5.4 *μ*m, subcylindrical to cylindrical, smooth, thin-walled, acyanophilous, inamyloid, with oily contents.

*Specimen Examined.* India, Uttarakhand: Nainital, Bhowali, on log of* Quercus leucotrichophora *A. Camus, Sanyal 6679 (PUN), July 24, 2010.

*Remarks.* This species is characterized by being smooth to tuberculate, grayish white basidiocarp, clavate, constricted to moniliform cystidia, and subcylindrical to cylindrical basidiospores. Earlier, it has been reported from North America, Europe, Russia, and United Kingdom (http://www.mycobank.org). However, it is being reported for the first time from India.

**Figure 5:**
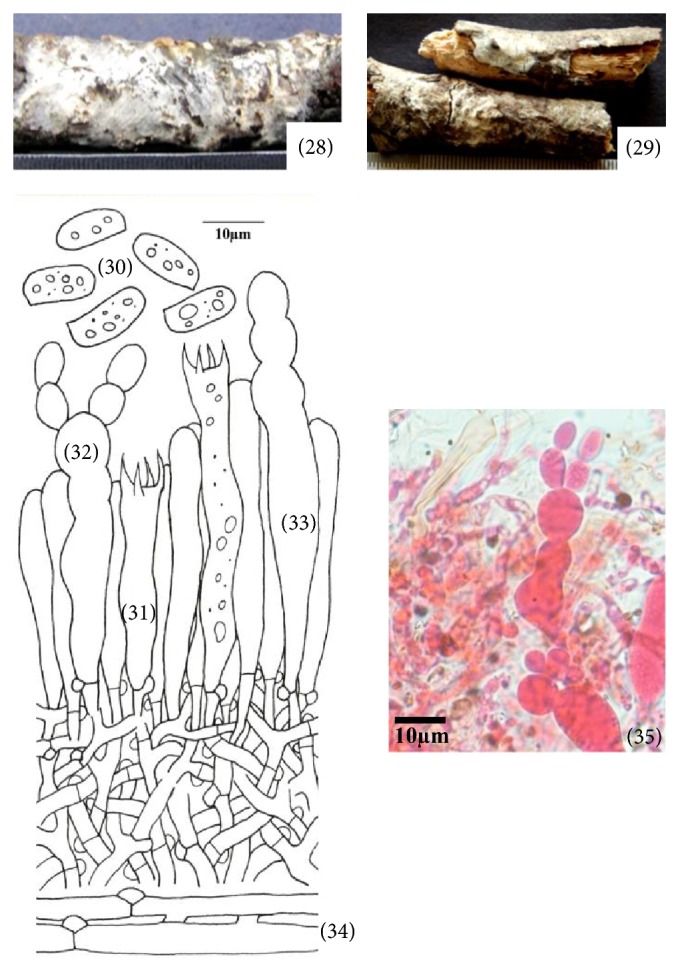
*Hyphoderma nemorale* (28)–(35). (28) Fresh basidiocarp, (29) dried basidiocarp, (30) basidiospores, (31) basidium, (32) monilicystidium, (33) cystidium, (34) generative hyphae, and (35) photomicrographs showing monilicystidium.

*(5) Hyphoderma nemorale K.H. Larss., Nordic Journal of Botany 18 (1): 123, 1998 (Figure 5(28)–(35)).* Basidiocarp resupinate, effused, adnate, up to 140 *μ*m thick in section; hymenial surface smooth, grayish white to yellowish white when fresh, not changing much on drying; margins thinning, pruinose, paler concolorous, to indeterminate. Hyphal system monomitic. Generative hyphae up to 4.4 *μ*m wide, branched, septate, clamped, thin-walled; basal hyphae parallel to the substrate, forming a loose texture; subhymenial hyphae vertical and denser. Cystidia of two types: (i) Leptocystidia 48.0–58.0 × 6.0–6.8 *μ*m, subcylindrical, thin-walled, with oily contents, resinous encrustation at the apex and a basal clamp. (ii) Moniliform cystidia 43.0–53.0 × 6.2–10.0 *μ*m, often moniliform with deep constrictions, sometimes branched at the tip, smooth, thin- to somewhat thick-walled, with basal clamp. Basidia 29.0–34.0 × 6.2–8.0 *μ*m, clavate, often constricted to somewhat sinuous, with oily contents, 4-sterigmate, with basal clamp; sterigmata up to 5.0 *μ*m long. Basidiospores 10.4–14.4 × 5.4–6.2 *μ*m, subcylindrical to ellipsoid, smooth, thin-walled, acyanophilous, inamyloid, with oily contents.

*Specimen Examined.* India, Uttarakhand: Nainital, Bhimtal, on stick of* Quercus leucotrichophora *A. Camus, Sanyal 6685 (PUN), July 24, 2010.

*Remarks.* This species is characterized by being smooth, grayish white to yellowish white basidiocarp, constricted, subcylindrical to moniliform cystidia along with leptocystidia, clavate constricted basidia, and subcylindrical to ellipsoid basidiospores. Larsson [32] described it for the first time from Switzerland. Later, it was reported from the Caucasus, Denmark, Finland, France, Germany, Norway, Italy, Romania, Russia, Spain, Sweden, Switzerland, and Ukraine (http://www.mycobank.org). However, it is being reported for the first time from India.

**Figure 6:**
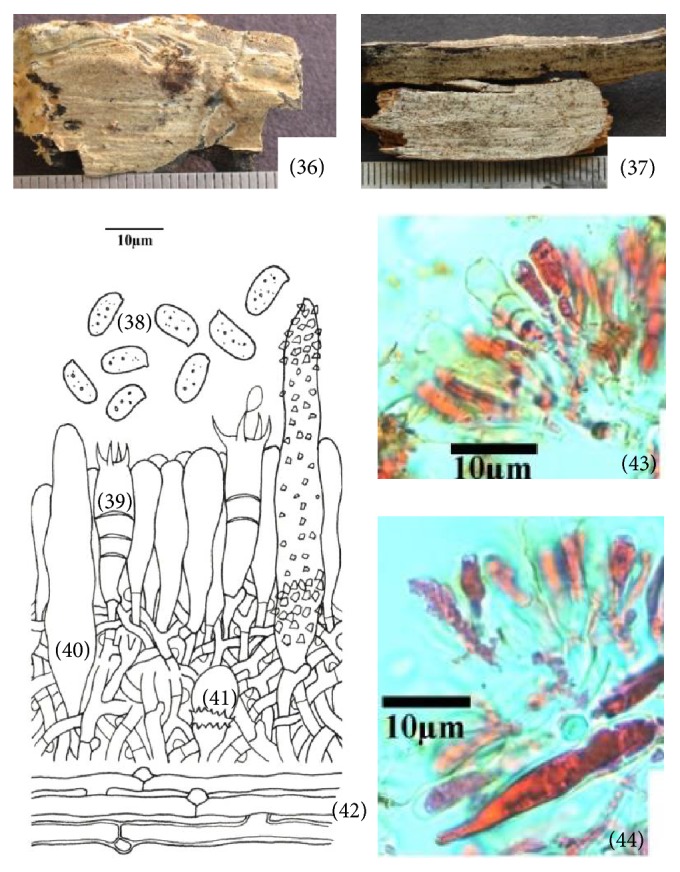
*Hyphoderma subpraetermissum* (36)–(44). (36) Fresh basidiocarp, (37) dried basidiocarp, (38) basidiospores, (39) basidium, (40) cystidium, (41) stephanocyst, (42) generative hyphae, (43) photomicrographs showing basidium, and (44) microphotographs showing cystidium.

*(6) Hyphoderma subpraetermissum Sheng H. Wu, Botanical Bulletin of the Academia Sinica (Taipei) 38: 68, 1997 (Figure 6(36)–(44)).* Basidiocarp resupinate, effused, adnate, up to 150 *μ*m thick in section; hymenial surface smooth to tuberculate, grayish white when fresh, whitish to pale creamish gray on drying; margins thinning, paler concolorous, to indeterminate. Hyphal system monomitic. Generative hyphae up to 4.4 *μ*m wide, branched, septate, clamped, thin- to somewhat thick-walled; basal hyphae running parallel to the substrate, loosely arranged; subhymenial hyphae vertical, densely united. Sterile structures of two types: (i) Cystidia 48.0–60.0 × 8.0–8.4 *μ*m, cylindrical or ventricose, sometimes tapering towards apex, thin- to somewhat thick-walled, encrusted; enclosed to projecting. (ii) Stephanocysts 7.8 × 7.5 *μ*m at apical portion, bladder shaped, surrounded by a whorl of small teeth, with basal clamp. Basidia 23.0–26.0 × 6.6–7.0 *μ*m, clavate to subclavate, frequently with secondary septa, 4-sterigmate, with basal clamp; sterigmata up to 5.2 *μ*m long. Basidiospores 7.0–8.8 × 3.6–4.4 *μ*m, ellipsoid, thin-walled, smooth, acyanophilous, inamyloid, with oily contents.

*Specimen Examined.* India, Uttarakhand: Almora, Sitoli, on log of* Pinus roxburghii *Sarg., Sanyal 6805 (PUN), August 29, 2011.

*Remarks.* This species is characterized by being smooth to tuberculate and grayish white basidiocarp and differs from* Hyphoderma praetermissum* (P. Karst.) J. Erikss. & Å. Strid in having smaller basidiospores. Wu [29] was the first to describe it from Taiwan. Here, it is being described as a new record for India.

**Figure 7:**
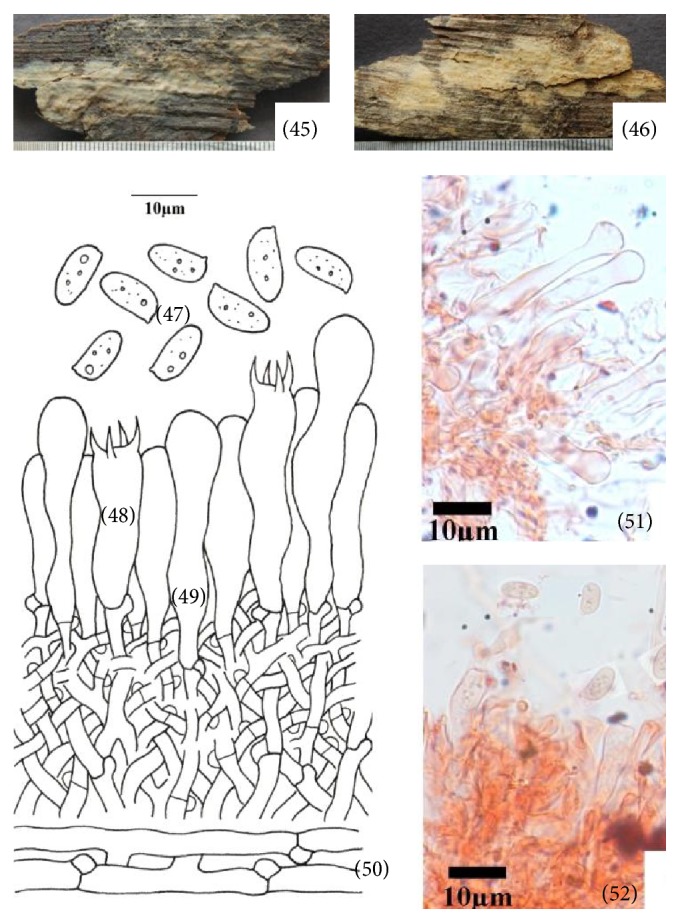
*Hyphoderma tibia* (45)–(52). (45) Fresh basidiocarp, (46) dried basidiocarp, (47) basidiospores, (48) basidium, (49) cystidium, (50) generative hyphae, (51) photomicrographs showing cystidium, and (52) photomicrographs showing basidiospores.

*(7) Hyphoderma tibia K.H. Larss., Grosse-Brauckm. & Jean Keller, Nordic Journal of Botany 18 (2): 239, 1998 (Figure 7(45)–(52)).* Basidiocarp resupinate, adnate, effused, up to 290 *μ*m thick in section; hymenial surface smooth to tuberculate, yellowish white to pale orange when fresh, pale yellow on drying, margins thinning, byssoid, paler concolorous, to indeterminate. Hyphal system monomitic. Generative hyphae septate, clamped, thin-walled; basal hyphae up to 5.0 *μ*m wide, less branched, parallel to substrate, loosely interwoven; subhymenial hyphae up to 3.0 *μ*m wide, much branched, vertical. Cystidia 22.0–43.0 × 5.0–6.8 *μ*m, cylindrical to subcylindrical, tibiform or subcapitate, thinp- to slightly thick-walled, with basal clamp; projecting up to 24.0 *μ*m out of the hymenium. Basidia 23.0–32.0 × 7.4–8.8 *μ*m, clavate to subclavate, 4-sterigmate, with basal clamp; sterigmata up to 5.0 *μ*m long. Basidiospores 8.8–9.4 × 3.8–5.0 *μ*m, ellipsoid to subcylindrical, somewhat tapering towards the apiculus, sometimes slightly concave at the adaxial side, thin-walled, smooth, acyanophilous, inamyloid, with oily contents.

*Specimen Examined.* India, Uttarakhand: Bageshwar, Jhandidhar, on log of* Pinus roxburghii *Sarg., Sanyal 6806 (PUN), September 02, 2011.

*Remarks.* This species is characterized by being yellowish white to pale orange basidiocarp, cylindrical to subcylindrical, tibiform or capitate cystidia and ellipsoid to subcylindrical, somewhat tapering towards the apiculus, sometimes slightly concave at the adaxial side, thin-walled basidiospores. This species was first described by Larsson [32] from Poland. Here, it is being described as a new record for India.

**Figure 8:**
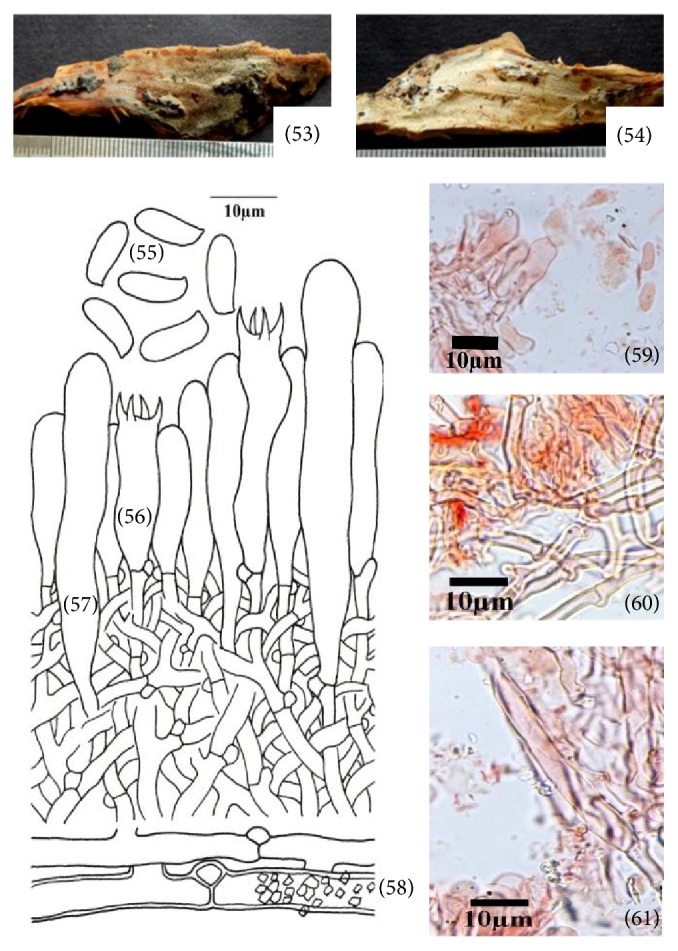
*Hyphoderma transiens* (53)–(61). (53) Fresh basidiocarp, (54) dried basidiocarp, (55) basidiospores, (56) basidium, (57) cystidium, (58) generative hyphae, (59) photomicrographs showing basidium and basidiospores, (60) photomicrographs showing generative hyphae, and (61) photomicrographs showing generative hyphae and cystidium.

*(8) Hyphoderma transiens (Bres.) Parmasto, Conspectus Systematis Corticiacearum: 114, 1968 – Odontia transiens Bres., Brotéria Série Botânica 11: 72, 1913 (Figure 8(53)–(61)).* Basidiocarp resupinate, adnate, effused, up to 150 *μ*m thick in section; hymenial surface odontoid, grayish white to pale orange when fresh, grayish white to orange white to brownish orange on drying; margins thinning, byssoid, paler concolorous, to indeterminate. Hyphal system monomitic. Generative hyphae septate, clamped; basal hyphae up to 5.4 *μ*m wide, less branched, parallel to substrate, thin- to thick-walled, loosely interwoven, encrusted; subhymenial hyphae up to 3.4 *μ*m wide, much branched, vertical, thin-walled, compact. Cystidia 63.0–76.0 × 9.4–11.8 *μ*m, subcylindrical to cylindrical, sinuous, apically widened, thin-walled, with basal clamp; projecting up to 13.0 *μ*m out of the hymenium. Basidia 20.0–32.0 × 6.2–7.6 *μ*m, clavate to subclavate, constricted, 4-sterigmate, with basal clamp; sterigmata up to 4.0 *μ*m long. Basidiospores 9.6–10.6 × 3.4–4.2 *μ*m, ellipsoid to suballantoid, thin-walled, smooth, acyanophilous, inamyloid.

*Specimen Examined.* India, Uttarakhand: Bageshwer, Kausani, on log of* Pinus roxburghii *Sarg., Sanyal 6807 (PUN), September 03, 2011.

*Remarks. Hyphoderma transiens* is characterized by odontoid hymenophore, subcylindrical to cylindrical, apically widened cystidia, clavate to subclavate, constricted basidia and ellipsoid to suballantoid basidiospores. Bresadola [33] described it as* Odontia transiens* Bres. Parmasto [4] shifted it to genus* Hyphoderma*. It is widely distributed in the European countries (the Caucasus, Croatia, Estonia, France, Germany, Italy, Portugal, Russia, Spain, Sweden, Switzerland, Turkey, and Ukraine) and United Kingdom (http://www.mycobank.org). However, it is being described for the first time from India.

## 5. Key to the Species

 See Table 1.

## 6. Conclusion

*Hyphoderma* Wallr. is a wood-decaying corticoid fungal genus and reported worldwide from tropical to temperate regions. Taxonomic and molecular studies in recent times have shown that it is widespread with a lot of morphological variability. Macro- and microscopic characteristics are usually reliable for the identification of* Hyphoderma* species. Exploration of the new areas in India will still add to the diversity of the species in this genus. This paper provides important information on new* Hyphoderma* records from India. All the reported species have been collected from the state of Uttarakhand and key to all the species reported from Uttarakhand is provided.

## Figures and Tables

**Table 1:** 

1	Dendrohyphidia present	*H*. *rimosum*
1	Dendrohyphidia absent	2
2	Cystidia absent	3
2	Cystidia present	4
3	Basidiospores longer than 10.0 *μ*m	*H*. *cremeoalbum*
3	Basidiospores up to 10.0 *μ*m	*H*. *sibiricum*
4	Sterile structures of one kind	5
4	Sterile structures of more than one kind	20
5	Cystidia longer than 100 *μ*m	6
5	Cystidia shorter than 100 *μ*m	10
6	Septate, clamped cystidia present	*H*. *setigerum*
6	Septate, clamped cystidia absent	7
7	Cystidia with reddish brown globule at apex	*H*. *guttuliferum*
7	Not as above	8
8	Basidiospores 10–12 *μ*m long	*H*. *medioburiense*
8	Basidiospores up to 8 *μ*m long	9
9	Basidiospores 6.2–8.0 × 2.4–3.8 *μ*m, ellipsoid	*H*. *clavigerum*
9	Basidiospores 4.4–5.6 × 2.4–3.0 *μ*m, subcylindrical to ellipsoid	*H*. *macedonicum*
10	Cystidia capitate to tibiform	*H*. *tibia*
10	Not as above	11
11	Heavily encrusted lamprocystidia present	*H*. *sporulosum*
11	Heavily encrusted lamprocystidia absent	12
12	Hymenial surface odontoid	*H*. *transiens*
12	Hymenial surface not odontoid	13
13	Cystidia moniliform	*H*. *litschaueri*
13	Not as above	14
14	Basidiospores broadly ellipsoid to ovoid to subglobose	15
14	Basidiospores ellipsoid to cylindrical	16
15	Cystidia basally widened, narrowing towards apex	*H*. *argillaceum*
15	Cystidia apically widened, narrowing towards base	*H*. *obtusum*
16	Basidiocarps with a dull rose tint	*H*. *roseocremeum*
16	Not as above	17
17	Cystidia enclosed, basidiospores ellipsoid	18
17	Cystidia projecting, basidiospores cylindrical to allantoid	19
18	Basidiospores 6.2–8.0 × 2.4–3.8 *μ*m	*H*. *clavatum*
18	Basidiospores 8.0–11.0 × 5.4–7.0 *μ*m	*H*. *lapponicum*
19	Basal hyphae encrusted, basidiospores 3.8–4.0 *μ*m wide	*H*. *definitum*
19	Basal hyphae smooth, basidiospores 4.4–5.0 *μ*m wide	*H*. *occidentale*
20	Septate, clamped cystidia along with cylindrical cystidia	*H*. *setigerum *var. *bicystidium*
20	Septate, clamped cystidia absent but other kinds of cystidia present	21
21	Moniliform cystidia along with subcapitate cystidia present	*H*. *nemorale*
21	Moniliform cystidia absent	22
22	Heavily encrusted cystidia along with gloeocystidia present	*H*. *puberum*
22	Heavily encrusted cystidia absent	23
23	Fusiform cystidia with capitate cystidia present	*H*. *pallidum*
23	Not as above	24
24	Echinocysts present	*H*. *echinocystis*
24	Stepahanocysts present	25
25	Basidia with retraction septa, basidiospores 7.4–8.4 × 3.6–4.4 *μ*m	*H*. *subpraetermissum*
25	Basidia without retraction septa, basidiospores 8.0–10.8 × 3.0–4.4 *μ*m	*H*. *praetermissum*
